# Short-term outcomes of laparoscopic central hepatectomy: a comparison with open surgery

**DOI:** 10.1007/s00423-025-03645-4

**Published:** 2025-02-20

**Authors:** Takashi Masuda, Yuichi Endo, Shun Nakamura, Wataru Miyoshino, Yuiko Nagasawa, Hiroki Orimoto, Masahiro Kawamura, Atsuro Fujinaga, Hiroomi Takayama, Yoko Kawano, Teijiro Hirashita, Masafumi Inomata

**Affiliations:** Department of Gastroenterological and Pediatric Surgery, Oita University Faculty of Medicine, Idaigaoka 1-1, Hasama-machi, Oita, 879-5593 Japan

**Keywords:** Laparoscopic central hepatectomy, Laparoscopic anterior sectionectomy, Laparoscopic central bisectionectomy, Bile leakage

## Abstract

**Purpose:**

Laparoscopic liver resection has advantages over open liver resection, including reduced blood loss and shorter hospital stays. Laparoscopic central hepatectomy (CH) is a highly challenging procedure, and such outcomes can only be achieved with a high level of expertise in laparoscopic liver surgery. Laparoscopic CH remains challenging, with safety and efficacy unclear. This study retrospectively evaluated the intra- and short-term outcomes of laparoscopic versus open CH and assessed safety and efficacy of laparoscopic surgery.

**Methods:**

CH included anterior sectionectomy (AS) and central bisectionectomy (CBS) (excluding cases with biliary reconstruction). The study comprised 38 patients receiving CH in our department from January 2010 to November 2023.

**Results:**

The laparoscopic group included 14 cases and the open group 24. AS/CBS was performed in 14/10 open group cases and 6/8 laparoscopic group cases. Short-term surgical outcomes showed no significant between-group difference in operative time, but the laparoscopic group lost significantly less blood (250 vs. 985 mL; *p* = 0.001) and the transfusion rate was lower (14% vs. 46%; *p* = 0.004). Among postoperative outcomes, incidence of bile leakage was not significantly different (33% vs. 42%; *p* = 0.42), but the laparoscopic group had significantly less postoperative ascites (0% vs. 17%; *p* = 0.047), fewer surgical site infections (SSI) (0% vs. 21%; *p* = 0.02), and shorter postoperative hospital stay (14 vs. 30 days; *p* = 0.005). The risk factor for bile leakage in CH by univariate and multivariate analysis was tumor proximity to the right anterior Glissonean pedicle (OR = 6.84; 95% CI = 1.67–32.7; *p* = 0.01).

**Conclusion:**

Laparoscopic CH could be performed safely. Compared to open surgery, laparoscopic CH was effective in reducing blood loss, postoperative ascites, SSIs, and shortening postoperative hospital stay. However, tumors close to the root of the right anterior Glissonean pedicle were considered a risk factor for postoperative bile leakage and require caution.

**Supplementary Information:**

The online version contains supplementary material available at 10.1007/s00423-025-03645-4.

## Introduction

Laparoscopic liver resection (LLR) was first reported in 1991 by Reich et al. [1]. With the rapid advancement of laparoscopic techniques and equipment, the rate of LLR has been increasing worldwide, and the indications for LLR have expanded [2–4]. Increasing numbers of authors have reported the efficacy of LLR, which is associated with reduced blood loss, lower transfusion requirements, and shorter length of hospital stay [5–8].

Centrally located tumors (in segments 4, 5, and 8) require central hepatectomy (CH), which involves central bisectionectomy (CBS) or right anterior sectionectomy (AS). Although CH has the advantage of preserving sufficient remnant liver volume, it involves two liver dissection planes, and the surgical procedure is therefore complicated. Thus, CH has been reported as a surgical procedure associated with a high risk of bile leakage due to exposure of major Glissonean structures and the extensive liver dissection planes [9–11]. There have been some recent reports on laparoscopic CH (LCH) [12–17], although LCH carries a high degree of difficulty [18–20]. LCH can only be achieved with a high level of expertise in laparoscopic liver surgery. Further, the safety and feasibility of this procedure have not been established yet.

Therefore, the aim of this study was to retrospectively evaluate the intra- and short-term outcomes of laparoscopic vs. open CH cases in our department and assess the safety and efficacy of LCH.

## Materials and methods

### Patients and methods

CH was defined as CBS and AS requiring resection of the right anterior Glissonean pedicle (excluding cases with biliary reconstruction). In total, 38 patients underwent CH at the Department of Gastroenterological and Pediatric Surgery, Oita University Faculty of Medicine between January 2010 to November 2023. At our institution, we perform approximately 60 liver resections annually, with laparoscopic procedures accounting for about 80% of them. Our team includes board-certified surgeons recognized by the Japanese Society of Hepato-Biliary-Pancreatic Surgery for advanced skills and by the Japan Society for Endoscopic Surgery for expertise in laparoscopic liver resection, thus ensuring high-quality surgical outcomes. We collected information on age, sex, body mass index (BMI), tumor characteristics, tumor proximity (< 1 cm) to the hepatic vein or the root of the right anterior Glissonean pedicle, clinical data, histopathology, surgical methods, operative time, blood loss, length of postoperative hospital stay, and postoperative complications from the patients’ medical records. We retrospectively compared these data between the open CH (OCH) and LCH groups to assess the safety and efficacy of laparoscopic surgery.

This study was approved by the Ethics Committee of Oita University Faculty of Medicine (No. 1601). Informed consent was obtained in the form of opt-out on the hospital web site.

### Surgical procedure

A video of our standardized laparoscopic CBS procedure is presented in Video 1. In all laparoscopic cases, the operations were started with the patient in the left half-lateral decubitus position, and the first port was inserted through a supraumbilical incision by the open method. Four other ports were inserted in the right upper quadrant of the abdomen. At our institution, liver resection is performed with the intermittent Pringle maneuver in all cases. First, after cholecystectomy, the right anterior Glissonean pedicle is secured, and a clamp test is performed to confirm the demarcation line. For AS, the cranioventral approach is used to dissect the liver along the middle hepatic vein from its root toward the root of the right anterior Glissonean pedicle. For CBS, the left transection plane between segment 4 and the left lateral lobe is dissected along the falciform ligament on the surface of the liver. Then, branches of segment 4b and segment 4a are ligated and gradually transected. Dissection is continued toward the top of the left plane, and the roots of the left hepatic and middle hepatic veins are exposed. At the same time, at the hepatic hilum, dissection between segment 4 and the caudate lobes is advanced along the hepatic hilar plate toward the root of the right anterior Glissonean pedicle. After dissection around the root of the right anterior Glissonean pedicle is fully developed, the right anterior Glissonean pedicle is dissected with a stapling device. Finally, the liver is dissected in one direction toward the right side so as to connect the left wall of the right hepatic vein and the demarcation line on the surface, which then completes the dissection.

### Statistical analysis

Continuous data are expressed as the median value and range (lowest and highest), and categorical data are expressed as counts, with the associated percentile value calculated. The Wilcoxon rank sum test was used to compare continuous data, and Pearson’s χ^2^ test was used for categorical data. A multivariate analysis was performed using a Cox proportional hazard model to identify independent risk factors of bile leakage after CH. A p-value < 0.05 was considered to indicate statistical significance. Multivariate analysis was performed for factors with a p-value < 0.10 on univariate analysis. All statistical analyses were performed with JMP software version 17.2 for MAC (SAS Institute, Cary, NC, USA).

## Results

### Comparison of patient clinical characteristics

Clinical characteristics of the patients can be compared between the OCH and LCH groups in Table 1. There were no differences in tumor condition and liver function between the two groups, although there were a greater number of females in the LCH group.

**Table 1:** Patient characteristics

Characteristic	OCH	LCH	*p* Value
	(*n* = 24)	(*n* = 14)	
Age	74 (50–83)	76.5 (61–89)	0.4
Gender (male/female), n (%)	19 (79)/5 (21)	6 (43)/8 (57)	0.02
BMI (kg/m^2^)	23.4 (19.8–32.2)	23.2 (17.8–32.9)	0.53
ASA-PS (1–2/3), n (%)	17 (71)/7 (29)	11 (79)/3 (21)	0.6
Diagnosis (HCC/CRLM/IHCC/Other), n (%)	15 (63)/6 (25)/2 (8.3)/1 (4.2)	11 (79)/2 (14)/0/1 (7.1)	0.42
Tumor condition			
Maximal diameter of tumor (mm)	56.5 (13–140)	41 (5-100)	0.09
Number of tumors	1 (1–6)	1 (1–3)	0.36
Tumor proximity to the root of the anterior Glissonean pedicle, n (%)	11 (46)	3 (21)	0.12
Tumor proximity to the hepatic vein, n (%)	11 (46)	6 (42)	0.86
Liver function			
ICG-R15 (%)	11.9 (4.4–26.6)	7.8 (2.2–26.5)	0.06
Liver cirrhosis, n (%)	4 (17)	4 (31)	0.33

Legends: ASA-PS = American Society of Anesthesiologists physical status; BMI = body mass index; CRLM = colorectal liver metastasis; HCC = hepatocellular carcinoma; ICG = indocyanine green; IHCC = intrahepatic cholangiocarcinoma; LCH = laparoscopic central hepatectomy; OCH = open central hepatectomy

### Comparison of surgical outcomes

Surgical outcomes between the LCH and OCH groups can be compared in Table 2. There was one case of open conversion in the LCH group. There was no difference in operative time between the two groups, but the LCH group had significantly less blood loss and lower transfusion rates. The postoperative outcomes are shown in Table 3. There was no difference in the incidence of bile leakage between the two groups (33% vs. 42%; *p* = 0.42). The incidences of ascites (0% vs. 17%; *p* = 0.047) and surgical site infection (SSI) (0% vs. 21%; *p* = 0.02) were significantly lower in the LCH group. There were no significant differences in other complications. Postoperative hospital stay was significantly shorter in the LCH group (14 vs. 30 days; *p* = 0.005).

**Table 2:** Surgical outcomes

Outcome	OCH	LCH	*p* Value
	(*n* = 24)	(*n* = 14)	
Anterior sectionectomy/central bisectionectomy, n (%)	14 (58)/10 (42)	8 (57)/6 (43)	0.94
Open conversion, n (%)	-	1 (7.1)	-
Operative time (min)	396 (265–550)	353.5 (261–569)	0.08
Blood loss (mL)	985 (310–5420)	250 (90-1000)	< 0.001
Transfusion, n (%)	11 (46)	2 (14)	0.004

Legends: LCH = laparoscopic central hepatectomy; OCH = open central hepatectomy

**Table 3:** Postoperative outcomes

Outcome	OCH	LCH	*p* Value
	(*n* = 24)	(*n* = 14)	
Complications after surgery (CD ≥ III) n (%)	11 (46)	5 (36)	0.54
Bile leakage, n (%)	10 (42)	3 (33)	0.42
Abdominal abscess, n (%)	7 (29)	1 (7.1)	0.17
Liver failure, n (%)	2 (8.3)	0	0.25
Ascites, n (%)	4 (17)	0	0.047
Renal failure, n (%)	1 (4.2)	1 (7.1)	0.69
Surgical site infections, n (%)	5 (21)	0	0.02
Pleural effusion, n (%)	2 (8.3)	0	0.17
Cardiovascular complication, n (%)	0	1 (7.1)	0.15
Gastrointestinal complication, n (%)	0	1 (7.1)	0.15
Reintervention, n (%)	10 (42)	4 (29)	0.42
Reoperation, n (%)	0	0	-
Postoperative stay (days)	30 (11–293)	14 (7–53)	0.005

Legends: CD = Clavien-Dindo classificatisn; LCH = laparoscopic central hepatectomy; OCH = open central hepatectomy

### Risk factors for bile leakage in central hepatectomy

CH is a procedure associated with a high incidence of bile leakage, and thus the risk factors for bile leakage were investigated. Univariate and multivariate analysis both revealed proximity of the tumor to the right anterior Glissonean pedicle to be the significant risk factor for bile leakage (odds ratio, 6.84; 95% confidence interval, 1.67–32.7; *p* = 0.01)(Table 4).

**Table 4:** Risk factors for bile leakage in central hepatectomy

	Univariable			Multivariable		
	Odds ratio	95% CI	p Value	Odds ratio	95% CI	p Value
BMI (kg/m^2^) (≥ 25 vs. < 25)	2.42	0.62–9.54	0.2			
Number of tumors (≥ 2 vs. 1)	0.97	0.23–4.17	0.97			
Liver cirrhosis (Yes vs. No)	1.04	0.21–5.2	0.97			
Tumor size (≥ 50 mm vs. < 50 mm)	1.4	0.37–5.27	0.62			
Tumor proximity to the root of the anterior Glissonean pedicle (Yes vs. No)	6.84	1.57–29.79	0.007	6.84	1.67–32.7	0.01
Tumor proximity to the hepatic vein (Yes vs. No)	0.56	0.14–2.15	0.39			
Laparoscopy vs. Open	0.56	0.14–2.3	0.42			
Anterior sectionectomy vs. central bisectionectomy	1.05	0.27–3.99	0.94			

Legends: BMI = body mass index, CI = confidence interval

## Discussion

In this study, we retrospectively reviewed cases of LCH and OCH performed in our department to assess the safety and efficacy of laparoscopic surgery. Compared to the OCH group, the LCH group showed significantly less blood loss, lower transfusion rate, and no difference in operative time. Among postoperative complications, the rate of bile leakage was 33% in the LCH group and 42% in the OCH group, but the difference was not significant. The incidences of ascites and SSI were significantly lower in the LCH group, and postoperative hospital stay was significantly shorter in the LCH group. Univariate and multivariate analysis of risk factors for bile leakage after CH showed tumor proximity of < 1 cm to the right anterior Glissonean pedicle to be the significant risk factor.

LCH is a highly complex procedure that requires extensive training [18–20]. At our institution, we establish surgeon qualification criteria based on a scoring system for LLR [18, 19]. We begin with partial resections for low-difficulty anterolateral lesions and progressively advance to more complex liver resections in a step-by-step manner. To perform major hepatectomy such as LCH, we believe that, as reported by Kuemmerli et al. [21] and Hasegawa et al. [22], experience with at least 60 cases of LLR is essential. While studies have investigated international benchmarks for the outcomes of LLR [23], no such benchmarks have been established for LCH. Other previous studies have reported the outcomes of LCH compared to OCH [15–17]. Among the short-term surgical outcomes, there was no difference in blood loss and the incidence of postoperative complications between the LCH and OCH groups, whereas the LCH group had a longer operative time and shorter postoperative hospital stay [15–17]. Cho et al. reported an overall postoperative morbidity rate of 30% and a bile leakage rate of 15% in their LCH group [15]. In our study as well, the LCH group experienced less blood loss, lower incidences of ascites and SSI, and a shorter postoperative hospital stay, consistent with these previous reports. However, the incidence of postoperative bile leakage in our patients was higher than that previously reported, so we investigated the risk factors for bile leakage. To our knowledge, there have been no reports investigating the causes of bile leakage in LCH, making the present findings novel in this respect.

In general, LLR is reported to be associated with fewer complications compared to open hepatectomy, but the incidence of bile leakage has been reported to be comparable when limited to CH [7, 8, 15, 24]. CH is considered to be a surgical procedure with a high incidence of bile leakage [9–11]. Nanashima et al. reported an incidence rate of bile leakage of 37% [10], whereas Ueno et al. reported a rate of 44.8% [25]. Several reports have examined the risk factors for bile leakage in CH [9, 10, 25]. Ueno et al. examined the biliary complications in CH and reported that the tumor proximity to the right anterior Glissonean pedicle and a longer right hepatic duct were risk factors for biliary complications [25]. In our study as well, proximity of the tumor to the right anterior Glissonean pedicle was found to be the risk factor for bile leakage. This factor could be the potential cause for stenosis of the right posterior bile duct (RPBD) branch. Yoon et al. investigated the factors contributing to the occurrence of the RPBD stenosis in CH [26]. Approximately 80% of the RPBD is located supraportally distal to the bifurcation of the anterior and posterior Glissonean sheaths. When the anterior Glissonean sheath is ligated, injuries such as stricture and ischemic insult of the RPBD could occur. They reported that handling the Glissonean pedicle close to the right anterior Glissonean pedicle root could lead to stenosis of the RPBD. This stenosis increases the intraductal pressure in the RPBD, potentially causing bile leakage from the liver dissection plane. Additionally, in cases in which the tumor is closer to the hepatic hilum, there is a higher possibility of incising the caudate lobe and damaging its bile ducts. This is consistent with the findings of Nanashima et al., who reported that segment 1 resection and inferior vena cava compression were risk factors for bile leakage with CBS [10]. These may be the reasons why a tumor close to the root of the right anterior Glissonean pedicle were detected as risk factors for bile leakage.

Recently, the robotic approach has been increasingly adopted in liver surgery. Robotic-assisted liver resection (RLR) offers several advantages over LLR, including enhanced precision, reduced blood loss, lower conversion rates, and higher R0 resection rates [27, 28]. These benefits stem from the robotic system’s superior visualization, increased instrument dexterity, and improved maneuverability. However, the widespread adoption of RLR remains limited due to its higher costs, longer learning curve, and the lack of significant differences in long-term oncological outcomes compared to LLR [27, 28]. While its utility in complex liver resections, such as CH, appears promising, further studies are required to validate its advantages.

The present study has several limitations. First, this is a single-center study with a small number of cases. Second, the operator was different in each case although the surgical team included expert board-certified surgeons. Third, long-term prognoses were not comparable because of the variety of diseases in the patients included in this study.

## Conclusion

Compared to OCH, LCH reduced blood loss and could be performed safely. Although the frequency of postoperative bile leakage was not different between these two procedures, there was less postoperative ascites and fewer SSIs with LCH, suggesting the potential for a shortened postoperative hospital stay. However, proximity of the tumor to the root of the right anterior Glissonean pedicle was considered a risk factor for postoperative bile leakage, and caution is required in such cases.

## Electronic supplementary material

Below is the link to the electronic supplementary material.

Supplementary Material 1

## Data Availability

No datasets were generated or analysed during the current study.
